# State Insurance

**Published:** 1911-07-22

**Authors:** Samuel S. Cole

**Affiliations:** Secretary. Royal Devon and Exeter Hospital, Exeter.


### INSTITUTIONAL LETTER-BOX.

### STATE INSURANCE.

*To the Editor of* The Hospital.

Sir,—I thank you for your letter of the 7th inst., together with copy of The Hospital, and I note that you are publishing a series of articles on the insurance system of other countries, which I shall follow with interest from time to time. My committee have had under consideration for some time the question of the National State Insurance, and on Friday last they decided to support the following resolution: "This meeting of the Incorporated Association of Hospital Officers, having considered the National Insurance Bill now before the House of Commons, is of opinion that provision should be made in the Bill for payment to be made to the hospitals in respect of all insured persons receiving treatment, whether as in or as out patients," which was recently passed at a meeting of the Incorporated Association of Hospital Officers held in London, and I was instructed to send a copy of the resolution to all the county and borough Members of Parliament throughout Devonshire, requesting them to be good enough to support any amendments to the Insurance Bill in the sense of the resolution.—Yours faithfully,

Samuel S. Cole, Secretary.

Royal Devon and Exeter Hospital, Exeter.
July 10, 1911.

### A NEW SIGN OF SCARLET FEVER.

*To the Editor of* The Hospital.

